# Mapping and Validation of the Major Sex-Determining Region in Nile Tilapia (*Oreochromis niloticus* L.) Using RAD Sequencing

**DOI:** 10.1371/journal.pone.0068389

**Published:** 2013-07-11

**Authors:** Christos Palaiokostas, Michaël Bekaert, Mohd G. Q. Khan, John B. Taggart, Karim Gharbi, Brendan J. McAndrew, David J. Penman

**Affiliations:** 1 Institute of Aquaculture, School of Natural Sciences, University of Stirling, Stirling, Scotland, United Kingdom; 2 Department of Fisheries Biology and Genetics, Bangladesh Agricultural University, Mymensingh, Bangladesh; 3 The GenePool, School of Biological Sciences, University of Edinburgh, Edinburgh, Scotland, United Kingdom; Temasek Life Sciences Laboratory, Singapore

## Abstract

Sex in *Oreochromis niloticus* (Nile tilapia) is principally determined by an XX/XY locus but other genetic and environmental factors also influence sex ratio. Restriction Associated DNA (RAD) sequencing was used in two families derived from crossing XY males with females from an isogenic clonal line, in order to identify Single Nucleotide Polymorphisms (SNPs) and map the sex-determining region(s). We constructed a linkage map with 3,802 SNPs, which corresponded to 3,280 informative markers, and identified a major sex-determining region on linkage group 1, explaining nearly 96% of the phenotypic variance. This sex-determining region was mapped in a 2 cM interval, corresponding to approximately 1.2 Mb in the *O. niloticus* draft genome. In order to validate this, a diverse family (4 families; 96 individuals in total) and population (40 broodstock individuals) test panel were genotyped for five of the SNPs showing the highest association with phenotypic sex. From the expanded data set, SNPs *Oni*23063 and *Oni*28137 showed the highest association, which persisted both in the case of family and population data. Across the entire dataset all females were found to be homozygous for these two SNPs. Males were heterozygous, with the exception of five individuals in the population and two in the family dataset. These fish possessed the homozygous genotype expected of females. Progeny sex ratios (over 95% females) from two of the males with the “female” genotype indicated that they were neomales (XX males). Sex reversal induced by elevated temperature during sexual differentiation also resulted in phenotypic males with the “female” genotype. This study narrows down the region containing the main sex-determining locus, and provides genetic markers tightly linked to this locus, with an association that persisted across the population. These markers will be of use in refining the production of genetically male *O. niloticus* for aquaculture.

## Introduction

Patterns of sex determination and differentiation in fish are very varied, with a wide range of gonochoristic and hermaphroditic species. Among the gonochoristic species, genetic and/or environmental factors determine sex [1]–[3]. The first sex-determining gene isolated in a fish species was *DMY/dmrt1bY* in *Oryzias latipes* (medaka) [4], [5]. More recently, several other fish sex-determining genes have been isolated: *Gsdf(Y)* in *Oryzias luzonensis* (Luzon ricefish) [6], [7]; *amhy* in *Odontesthes hatcheri* (Patagonian pejerrey) [2], [8]; *Amhr2* in *Takifugu rubripes* (tiger pufferfish) [1]; and *sdY* in *Oncorhynchus mykiss* (rainbow trout) [4]. Four of these genes come from those involved in sexual differentiation, while one derives from an immune-related gene (the *sdY* protein in *O. mykiss* is similar to part of the interferon regulatory factor 9). In all of these species the sex determination system is principally XX/XY, but mismatches between sexual genotype and phenotype are not uncommon [6], sex reversal can be induced, YY males are viable in several species, and differentiated sex chromosomes are relatively uncommon [2]. The consequences of such variation are that sex determination genes in fish may likely require identification at the species or genus level. Furthermore, while we might search for genes from the sex differentiation pathway in a region where a sex determination gene has been mapped, it is by no means certain that we can easily identify a master switch gene.

Since many farmed species of fish exhibit sexual dimorphism in a range of traits of interest like growth or age at maturity, clarification of the sex-determining system of such fish is beneficial for the aquaculture industry towards the production of mono-sex stocks. *Oreochromis niloticus* (Nile tilapia) is one of the most important farmed species with a production exceeding 2.8 million metric tonnes in 2010 [9]. Intensive commercial production generally requires all-male stocks, not only because males grow faster but also to avoid uncontrolled reproduction before harvest.

In tilapias, evidence so far suggests the existence of two different major sex-determining systems. In some tilapia species, including *O. niloticus* and *Oreochromis mossambicus* (Mozambique tilapia), sex is primarily determined by an XX/XY system on linkage group (LG) 1, whereas in others, for example *Oreochromis aureus* (blue tilapia), sex is primarily determined by a WZ/ZZ system on LG 3 [10]. However, other factors may influence sex determination and differentiation. In *O. niloticus*, genes on LG 3 [11] and LG 23 [12], [13], and temperature [14] can affect sex ratio. Crosses between YY males and XX females generally give less than to 100% male progeny predicted from a simple XX/XY system [15]. Many of the studies on sex determination in *O. niloticus* have been carried out on fish derived from Lake Manzala in Egypt, the subject of the present study, and it is clear that both non-LG 1 genes and temperature affect sex ratios in at least some families in this population.

The current linkage map for *O. niloticus* is based on more than 500 markers, mostly microsatellites [16]. The sex-determining region has been previously mapped close to microsatellite *UNH995* on LG 1 [17]. This region contains two genes implicated in vertebrate sexual differentiation, wt*1b* and *cyp19a*, but further mapping ruled these out as candidates for the major sex-determining locus [18]. Restriction site associated DNA (RAD) sequencing [19] offers the possibility to construct much higher density linkage maps in a cost-efficient manner. In this study we used RAD sequencing to identify single nucleotide polymorphisms (SNPs) in two crosses between XY males and females from an isogenic clonal line. A genetic map was constructed based on 3,802 SNP markers. A quantitative trait locus (QTL) analysis was conducted based on these SNPs and was followed by an association analysis for the SNPs that showed the highest association with phenotypic sex using a diverse dataset of both family and population data. Altogether these data located the sex-determining QTL in a region of approximately 1.2 Mb on LG 1.

## Materials and Methods

### Sample Collection and Preparation

The fish used in this study came from the Tropical Aquarium Facilities of the Institute of Aquaculture at the University of Stirling. They originated from a population that was established in 1979 from Lake Manzala, Egypt (31°16′N, 32°12′E). All working procedures complied with the Animals Scientific Procedures Act [20]. Fish were reared in recirculating water systems at 27–28°C, and fed on commercial trout diet (Trouw Aquaculture Nutrition, UK; manufacturer Skretting, UK). To set up the families used in this study, mature females were held in glass aquaria and eggs were manually stripped following ovulation. Milt was manually stripped from male fish and used to fertilise the eggs *in vitro*. Eggs were incubated in downwelling incubators until the larvae had absorbed the yolk sac. Fry from families 1–6 (Table 1) were then transferred to tanks in recirculating systems and reared for 3–4 months before being killed and sexed by microscopic examination of the gonads [21]. A sample of fin tissue was taken and fixed in 100% ethanol for DNA extraction. Family 7 was split at yolk sac absorption: one group of 80 fry was reared at 36°C for ten days [22] in a static 5 L tank, while a control group (80 fry) was reared at 28°C. The survival of the two groups was 88% and 91% respectively. Subsequent rearing and sexing was as for families 1–6.

**Table 1:** Summary of fish used in the study.

ID	Use	Sire strain	Dam strain	No. of females	No. of males	Total
Family1	RAD Libraries	Red†	Clonal	33	35	68
Family2	RAD Libraries	Red†	Clonal	10	10	20
Family3	SNP Assays	Red†	Wild*	12	12	24
Family4	SNP Assays	Red†	Wild*	12	12	24
Family5	SNP Assays	Wild*	Clonal	12	12	24
Family6	SNP Assays	Red†	Clonal	12	12	24
Family7 (28°C)	SNP Assays	Red†	Wild*	34	33	67
Family7 (36°C)	SNP Assays	Red†	Wild*	4	66	70
Clonal line	SNP Assays	–	–	0	2	2
Red strain	SNP Assays	–	–	6	15	19
Wild strain	SNP Assays	–	–	12	5	17

* “wild” refers to wild type coloration;

† “red” refers to red body colour, which is controlled by a single gene.

Families 1 and 2 (68 offspring and 20 offspring respectively) with dams from an isogenic XX clonal line and XY sires (as judged from balanced sex ratios in crosses to clonal line and outbred females and a high association between phenotypic sex and the LG 1 *UNH995* marker) were used to prepare RAD Libraries (Table 1). The available genome draft of *O. niloticus* is based on females from this clonal line. The sex associated SNPs were further validated by genotyping four further families with balanced sex ratios (Families 3–6: 24 offspring each) and broodstock (40 individuals; Table 1). These SNPs were finally used for genotyping a family in which elevated temperature induced a change in sex ratio (Family 7) to test whether the above SNPs could be useful in distinguishing neomales (XX males) from normal males. The sex ratio of the control group (reared at 28°C) did not show any deviations from the expected 1∶1 ratio, while in the high temperature (36°C) treated group the proportion of males exceeded 96%.

### RAD Library Preparation and Sequencing

DNA was extracted from fin tissue of the fish using the REALPure genomic DNA extraction kit (Durviz S.L.) and treated with RNase to remove residual RNA from the sample. Each sample was quantified by spectrophotometry (Nanodrop) and quality assessed by agarose gel electrophoresis, and was finally diluted to a concentration of 50 ng/µL in 5 mmol/L Tris, pH 8.5. The RAD library preparation protocol followed essentially the methodology originally described in Baird et al. [19] and comprehensively detailed in Etter et al. [23], with the minor modifications described in Houston et al. [24]. The RAD specific P1 and P2 paired-end adapters and library amplification PCR primer sequences used in this study are detailed in Baxter et al. [25].

Each sample (0.72 µg parental DNA/0.24 µg offspring DNA) was digested at 37°C for 40 minutes with *Sbf*I (recognising the CCTGCA|GG motif) high fidelity restriction enzyme (New England Biolabs; NEB) using 6 U *Sbf*I per µg genomic DNA in 1× Reaction Buffer 4 (NEB) at a final concentration of c. 1 µg DNA per 50 µL reaction volume. The reactions (12 µL final volumes) were then heat inactivated at 65°C for 20 minutes. Individual specific P1 adapters, each with a unique 5 bp barcode (Table 1), were ligated to the *Sbf*I digested DNA at 22°C for 60 minutes by adding 1.8/0.6 µL 100 nmol/L P1 adapter, 0.45/0.15 µL 100 mmol/L rATP (Promega), 0.75/0.25 µL 10× Reaction Buffer 2 (NEB), 0.36/0.12 µL T4 ligase (NEB, 2 M U/mL) and reaction volumes made up to 45/15 µL with nuclease-free water for each parental/offspring sample. Following heat inactivation at 65°C for 20 minutes, the ligation reactions were slowly cooled to room temperature (over 1 hour) then combined in appropriate multiplex pools (Table S1). Shearing (Covaris S2 sonication) and initial size selection (250–550 bp) by agarose gel separation [24] was followed by gel purification, end repair, dA overhang addition, P2 paired-end adapter ligation, library amplification, exactly as in the original RAD protocol [19], [23]. A total of 150 µL of each amplified library (16–18 PCR cycles, library dependent) was size selected (c. 350–650 bp) by gel electrophoresis [24]. Following a final gel elution step into 20 µL EB buffer (MinElute Gel Purification Kit, Qiagen), the libraries were sent to The GenePool Genomics Facility at the University of Edinburgh, UK, for quality control and high-throughput sequencing. Libraries were accurately quantified by qPCR (Kapa Library) and run in two lanes of an Illumina HiSeq 2000, one run using 100 base paired-end reads, the other 100 base single reads (v3 chemistry). Raw reads were process using RTA 1.12.4.2 and Casava 1.6 (Illumina). The reads were deposited at the NCBI BioProject under the accession SRP017804.

### Genotyping RAD Alleles

Reads of low quality (score under 30, while the average quality score was 37), missing the restriction site or with ambiguous barcodes were discarded. Retained reads were sorted into loci and genotyped using Stacks software 0.9995 [26]. The likelihood-based SNP calling algorithm [27] implemented in Stacks evaluates each nucleotide position in every RAD-tag of all individuals, thereby differentiating true SNPs from sequencing errors. A minimum stack depth of at least 30 and a maximum of 2 mismatches were allowed in a locus in an individual and up to 1 mismatch between alleles. The pair-ends were assembled using Stacks and Velvet version 1.2.08 [28] and used to separate RAD-tag sequence with or without potential SNP but belonging to separate loci (duplication products). Polymorphic RAD-tags may contain more than one SNP, but the vast majority (over 99%) showed only two allelic versions; the very small proportion of RAD-tags with more than two alleles were excluded.

### Genetic Map Construction

The genetic map was constructed using R/Onemap [29] and TMAP [30]. The allocation of markers into linkage groups was conducted using R/Onemap. This package uses Hidden Markov Models (HMM) algorithms for outbred species while in parallel implements the methodology described in Wu et al. [31] for calculating the most probable linkage phase. Linkage groups were formed using a minimum LOD value of 6. TMAP was used to order the markers in every linkage group. By using an HMM maximum likelihood model and taking into account potential genotypic errors it reduces the tendency to erroneously derive oversized linkage groups, a phenomenon which is often observed in dense maps. Map distances were calculated in centiMorgans (cM) using the Kosambi mapping function (Table S2). The linkage group name (number) was subsequently matched with the Broad Institute of MIT and Harvard genome assembly Orenil1.1 (NCBI Assembly GCA_000188235.2). The genetic map was drawn and aligned using Genetic-Mapper v0.3 [32].

### QTL Mapping

The QTL analysis was performed using R/qtl [33]. With the dam originating from a clonal line and by inferring the most probable phase of the genetic markers of the sire the cross had the same properties as a backcross and was analysed as such. Initially existence of single QTLs was tested (R/qtl function: *scanone*). The model used for the analysis was based on interval mapping. The algorithm used considers the phenotype to follow a mixture of Bernoulli distributions and uses a form of the expectation-maximisation algorithm for obtaining maximum likelihood estimates [33]. Permutation tests (10,000 permutations) were conducted in order to correct for the multiple testing. A multidimensional approach towards QTL mapping was adopted by using models from R/qtl that accounted for the QTL in LG 1, for two-QTLs and for multiple-QTLs simultaneously (R/qtl functions: *makeqtl, addqtl*, *scantwo, fitqtl, stepwiseqtl*). With this approach greater power can be achieved in the analysis allowing for detection of QTLs that would be remained undetected in the one-dimensional approach above. Approximate Bayesian 95% density intervals were calculated. An approximate estimate of the phenotypic variance explained by the QTL is obtained from the following equation: 1–10^−2LOD/n^. It has to be stressed that while the estimated variance may be reasonable for additive QTL, problems can be caused in the case of linked QTL [33].

### SNP Assays

We designed SNP assays using the KASP genotyping system (KBioscience UK Ltd) for five SNPs (*Oni*20117, *Oni*61067, *Oni*23063, *Oni*28137, *Oni*22734, NCBI dbSNP accession 748775078, 748775079, 748775085, 748775081 and 748775082 respectively; Table S3) that showed the highest association with sex in the two families that were used for the RAD-seq (Table S4). Allele-specific primers and other assay components were supplied by KBioscience UK Ltd, based on the supplied marker sequences (Table S3, Data S1). PCR reactions were carried for 10 µL final volume reactions. The cycling conditions were the following: 94°C for 15 min, 94°C for 20 sec, touchdown over 61°C to 55°C for 60 sec (10 cycles dropping 0.8°C each cycle) and an extra 34 cycles at 55°C.

### Association Analysis in Family and Population Data

Family data (offspring from four different families; 96 individuals in total) and 40 unrelated broodstocks (Table 1) were genotyped for the above five SNP markers. A family showing high response in elevated temperature was also genotyped for the same SNPs in order to check whether those SNPs could be used in distinguishing neomales from normal males. An association analysis was performed using R/SNPassoc [34]. In the case of family data, association was tested both in separate families and across all families together. A Bernoulli generalised linear model was applied in order to test the magnitude of association between the SNP genotypes and phenotypic sex using this package (function *association*). Both the Bonferroni and permutation tests (10,000 permutations) were used in order to correct for multiple testing.

## Results

### RAD Sequencing

**Figure 1:**
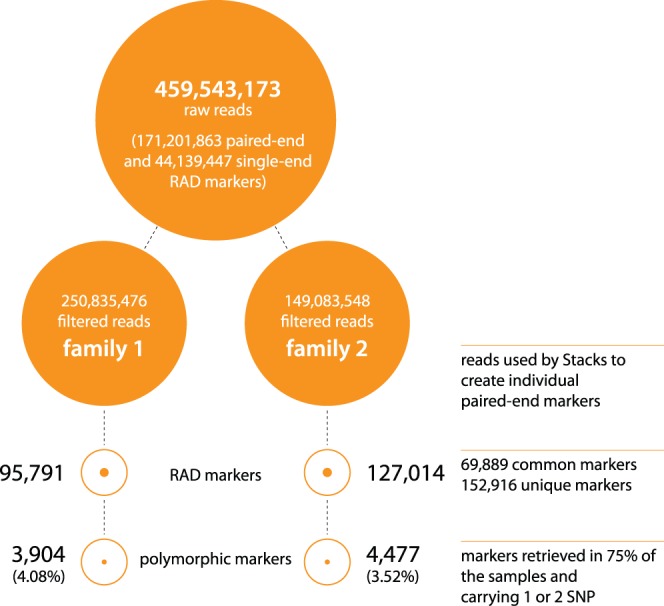
Sequencing and RAD-tag summary. Details of the number of reads before and after filters (orange disk) followed by the reconstructed number of RAD markers and polymorphic RAD markers (orange circles).

Two crosses with 68 and 20 offspring and their parents (including clonal females) were sampled (Table S1). The samples were barcoded, pooled and sequenced in two lanes of an Illuminia HiSeq 2000 sequencer (see Materials and Methods). In total, 459,543,173 raw reads (101 bases long) were produced (171,201,863 paired-end and 117,139,447 single-end reads, NCBI BioProject SRP017804). After removing low quality sequences (quality score under 30), ambiguous barcodes and orphaned paired-end reads, 87.03% of the raw reads were retained (399,918,024 reads). The Stacks package [26] was then used to make the assembly of the sampled loci from each individual: 95,791 and 127,014 RAD-tags were retrieved for Families 1 and 2 respectively, covering 152,916 RAD-tags in total including 69,889 of these shared between the two families (Figure 1). The number of reads and RAD-tags for each sample are reported in Table S1.

### Genetic Map

**Figure 2:**
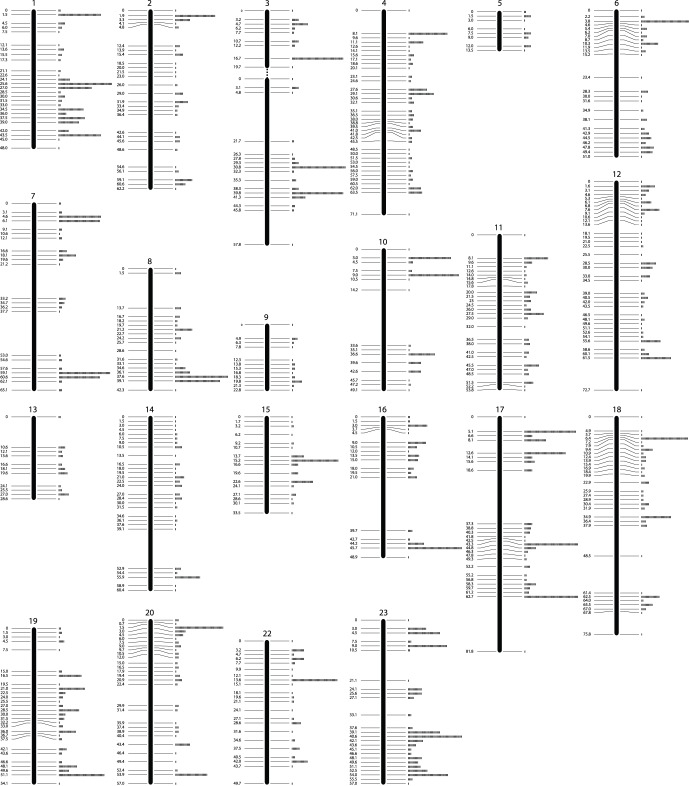
Genetic linkage map. Map with linkage group assignment determined using syntenic markers with previously published *O. niloticus* maps. The positions on the left side of the chromosomes are in cM. The rectangles on the right hand side represent the number of markers at this position. The numbering of the linkage groups corresponds to that in the Broad Institute genome anchored assembly Orenil1.1 [16], [47]. Detailed data are provided in Table S2.

In order to maximise the number of informative markers and minimise the amount of missing or erroneous data, we used SNP markers retrieved in at least 75% of the samples in each family, and carrying one or two SNPs. Since Family 2 had only 20 offspring, the genetic map was constructed with the Family 1 data only (68 offspring), while Family 2 was used to validate this. The sire-based map consists of 3,802 SNP markers that were grouped in 23 linkage groups, with an average spacing of 0.7 cM and spanning a total distance of 1,176 cM (Figure 2 and Tables 2 & S2). A unique genotypic pattern was observed in 3,280 of the above markers (522 showed identical inheritance patterns to other SNPs). In the second family (Family 2, 20 offspring) 724 of the above markers were heterozygous. The linkage groups were named according to the Broad Institute of MIT and Harvard genome assembly Orenil1.1 (NCBI Assembly GCA_000188235.2). We were not able to join the markers into 22 linkage groups corresponding to the 22 chromosomes expected from the karyotype [35]. However, by comparing our map to the draft tilapia genome sequence, two linkage groups were coalesced to form LG 3 in Figure 2. LG 3 contains a broad region of recombination suppression [36].

**Table 2:** *O. niloticus* Genetic Map.

LinkageGroup	No. ofmarkers	No. ofinformative markers	Length(cM)
1	284	248	48.0
2	136	123	62.2
3a	109	96	19.7
3b	153	138	57.8
4	191	177	71.1
5	27	25	13.5
6	161	134	51.0
7	232	197	65.2
8	170	136	42.3
9	32	32	22.8
10	143	111	49.1
11	135	119	53.8
12	173	155	72.7
13	47	41	28.6
14	90	86	60.4
15	192	157	33.5
16	154	124	48.9
17	323	277	81.5
18	188	160	75.8
19	246	210	54.1
20	155	136	57.0
22	117	111	49.7
23	344	287	57.0
Total	3,802	3,280	1,176

### QTL Mapping

**Figure 3:**
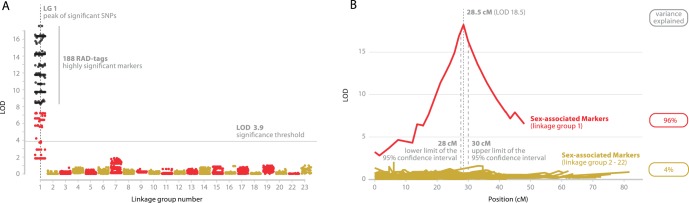
Results from QTL-Analysis. (A) Association results for genotyped SNPs. SNPs with *P*-values achieving genome-wide significance (*p*<7.2×10^−8^) are shown in black. (B) Regional analysis of the QTL. Plot of the LOD score (sex-association QTL search) along the linkage groups.

The results from the single-QTL model for binary traits provided evidence for the existence of a major QTL in LG 1 for both families (Figure 3A). The highest logarithm of odds (LOD) score for Families 1 and 2 were 18.50 and 6.02 respectively and the QTL was observed in the same location for both families with the same SNPs showing significant linkage (Figure 3B). The difference in the LOD scores reflects the smaller number of meioses in the second family (20 offspring). The fact that the marker phase was the same in the two families (in the QTL region) allowed an additional joint analysis of the two families. The genome wide threshold LOD value (*α* = 0.001) was 3.89 as calculated from permutation tests (10,000 permutations). The highest LOD score was observed at the 28.5^th^ cM of LG 1. Two adjacent SNPs were located in this position (*Oni*23063, *Oni*68581, from paired RAD-tags on either side of the same *Sbf*I restriction site). Only *Oni*23063 was analysed subsequently. The two-QTL model did not reveal any significant additional QTL or any evidence for epistasis. Even though models that take into account the existence of a major QTL (as in this study) or ones that test for existence of multiple QTLs simultaneously reduce the residual variation (providing higher power in the analysis for detecting additional QTLs at least of modest effect), no additional QTLs were detected. The calculated 95% Bayesian Density Intervals for the QTL location spanned a region of 2 cM (28–30 cM in LG 1), corresponding to approximately 1.2 Mb in the *O. niloticus* genome. The phenotypic variance explained by the QTL was 96%.

### Association Analysis using Family and Population Samples

**Figure 4:**
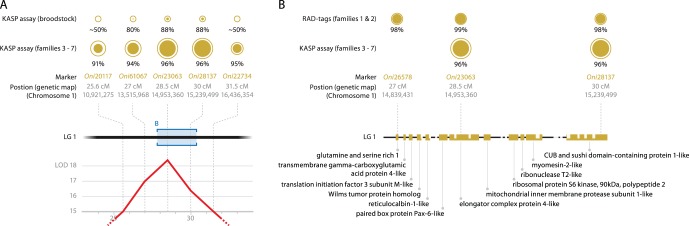
KASP assay and fine gene mapping on LG 1. (A) Details of the five markers tested by KASP assay. From bottom to top: LOD score (QTL sex-association); Location of the five markers (in the genetic map in cM and anchored draft genome in bp); KASP assay results. The outer circle diameters are proportional to the number of alleles tested. The inner (solid) disks represent the marker association with the phenotypic sex. Detailed data are provided in Table S4. (B) Details of the region of higher association. The bottom half is a schematic (to scale) of the chromosome LG 1, scaffold 17. It includes 14 gaps (white gaps) in the genome and 10 annotated genes (orange boxes). The sex-determining factor is located between “glutamine and serine rich 1” and “CUB and sushi domain-containing protein 1- like”. Detailed data are provided in Data S2.

The five SNP markers that showed the strongest linkage with sex (Figure 4A) in the two mapping families were tested in a larger panel consisting of family and population samples. The *p*-value thresholds (α = 0.05, multiple test correction) for permutation and the Bonferroni correction tests were 0.016 and 0.01 respectively. All five SNP markers were found to be significantly associated with sex in the family data both when tested in each family separately and across all families simultaneously (Figure 4A). For the population data (40 broodstock), four of the five SNP markers showed significant association with phenotypic sex (the exception was *Oni*20117, *p* = 0.73). *Oni*23063 and *Oni*28137 showed the highest association with phenotypic sex for both the population and family data (*p-*values of 1.08×10^−7^ and 3.024×10^−29^ respectively). Females were homozygous and males heterozygous for those two SNPs. The only exceptions from the above pattern were found for one male progeny from the four tested families and five male broodstock fish (Table S4). Progeny testing data were available for two of these five male broodstock (in crosses to XX females) and gave nearly all-female progeny (over 95% females), which would explain the homozygous SNP genotypes for these sires.

The sex-determining region spanned a distance of 2 cM, corresponding to approximately 1.2 Mb in the *O. niloticus* genome, with the peak (highest LOD score) located at the position of *Oni*23063 (Figure 3B). The two markers most strongly associated with sex, *Oni*28137 and *Oni*23063, are 400 kb apart in the *O. niloticus* genome. The 10 annotated genes within this region of the genome are therefore potential candidates for the sex-determining gene (Figure 4B and Data S2).

### Temperature Sex Reversal and Genotype

**Figure 5:**
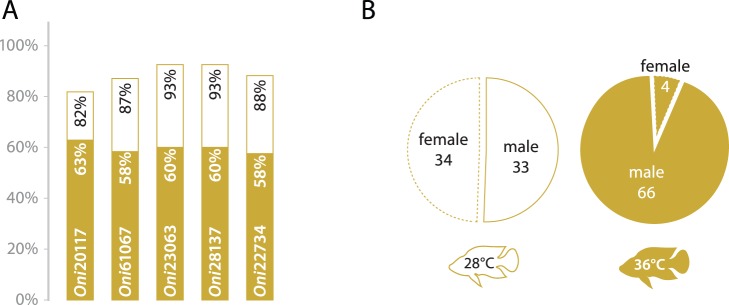
Sex reversion tests (family 7). (A) KASP assay results in family 7 offspring in response to elevated temperature (36°C) compare to standard 28°C. Each bar represent the ratio of male/female at 36°C (solid orange) and at 28°C (white background). (B) Detail of the phenotypic sex in response to elevated temperature (male have continuous border, female are presented with a discontinuous border).

To explore the effects of experimental sex reversal on the association between genotype and phenotypic sex, since family-specific QTLs involved in temperature sex determination have been recently identified [37], a family that showed a significant effect of raised temperature on sex ratio was genotyped for the two SNPs showing the strongest association (*Oni*23063 and *Oni*28137). While these showed highly significant association (*p* = 4.05×10^−14^ for both markers) in the control group, in the temperature treated group neither marker was significantly associated after correction for multiple testing (*p* = 0.039 for both markers). Five phenotypic males were found to deviate from the expected association in the control group, while in the treated group 28 phenotypic males deviated from the expected association for *Oni*23063 and *Oni*28137 (Figure 5).

## Discussion

Current evidence suggests that *O. niloticus* possess an XY/XX male heterogametic system complicated by genetic variance at this and other loci, environmental factors and probably by their interaction [36]. While previous family-based studies provided evidence for the existence of a major sex-determining region on LG 1 in *O. niloticus* [10], [17], [38], [39], various anomalies, including inconsistencies among families, have been observed. For example, Lee et al. [17] demonstrated the existence of a sex-determining region on LG 1, using microsatellite markers, which was mapped to an interval of 10 cM. However, the association between this region and phenotypic sex was only observed in two out of the three crosses studied. In the third cross, no association was observed with any genomic region. Frequent departures have been observed from the 100% male progeny in YY × XX crosses predicted from a single XX/XY locus [15], [40]. Eshel et al. [12], [13] found that markers in LG 23 showed the highest association with phenotypic sex in a cross in a population of *O. niloticus* also derived from Lake Manzala in Egypt. However no information was provided whether the above QTL on LG 23 persists in other crosses as well.

In the present study, our strategy was to use RAD sequencing to develop a much higher density linkage map than that of Lee et al. [16], based on carefully selected mapping families (isogenic female crossed to normal male; balanced sex ratio; progeny already tested for association between LG 1 microsatellite markers and phenotypic sex), then validate a set of tightly sex-linked markers in further family and population samples to test for population-wide association. SNP markers *Oni*61067, *Oni*23063 and *Oni*28137 showed the highest and most consistent association with phenotypic sex in all our data. *Oni*23063 was the marker with the highest LOD score in the two families that were used for the QTL analysis, while the additional families that were genotyped had the same magnitude of association with *Oni*28137, with no recombination observed between these two markers. *Oni*61067 gave the third largest association. The above provides evidence that the most probable location for the master sex-determining gene is in this region on LG 1, which spans around 1.2 Mb in scaffold 17 in the *O. niloticus* draft genome (Figure 4).

In all tested families, at each of the two loci (*Oni*23063 and *Oni*28137) females were homozygous for the same allele, while males were heterozygous, apart from two males (one each from Families 1 and 4) that were homozygous for the allele found in females. The results from the unrelated adult individuals showed again the same pattern, with the exception of five males that were homozygous for these two SNPs. Of these five, two were shown to give >95% female progeny in crosses to normal females (while no progeny testing data was available for the other three). No phenotypic female was mis-assigned in our entire dataset. Our data support the hypothesis that the mis-assigned (homozygous) males were in fact neomales, genetically female fish (as defined by the LG1 sex-determining locus) that have undergone sex reversal due to unknown genetic or environmental factor(s). The highly significant population-based linkage disequilibrium observed strongly supports the view that these two SNP markers are in very close proximity to the causative locus.

When a family responsive to sex reversal by exposure to elevated temperature was genotyped for *Oni*23063 and *Oni*28137, the temperature-treated group showed a significant number of phenotypic males with the female compatible genotype, while the typically strong association between SNP genotype and phenotypic sex was found in the control group. Rearing at high temperatures (36°C) results in significant masculinisation in some progenies if started around 10 days post fertilisation and applied for at least 10 days [41]–[43]. Wessels & Hörstgen-Schwark [22] showed that temperature-dependent sex ratio is a heritable trait. Lühmann et al. [37] mapped family-specific QTL for high temperature masculinisation effects to LG 1, LG 3 and LG 23. There is also evidence for feminisation by high temperature, from crosses expected to produce all-male progeny [44]–[46].

The genomic region, which was derived from the calculated density interval, includes 10 identified genes, none of which have yet been identified as the major sex-determining gene of any species. Nevertheless, it would not be prudent to exclude any of these as candidates at this time. A diversity of sex-determining systems has been documented in fish, implicating a variety of different, and in some cases unexpected, genes. For example, in *O. mykiss*, a gene associated with immune-related functions in other organisms was found to be its master sex-determining gene [4]. However, despite the fact that the results of this study showed that the major sex-determining factor is in close proximity with the tested SNPs, we cannot be confident that the major sex-determining gene is necessarily one of these ten genes. If the master sex-determining gene is male-specific (as found in *O. latipes* [3] and *O. mykiss* [7]), its presence will not be detectable from the current draft tilapia genome, which is derived from an isogenic female line. Finally there is also the possibility that the major sex-determining gene remains un-annotated in the current genome draft.

This study did not address the reasons why YY × XX crosses yield varying proportions of females. However, the SNP markers developed will help to tackle this issue, a key constraint on the commercial development of this technique.

## Conclusions

This study provides a linkage map of the *O. niloticus* genome that is several-fold denser than the existing one, a reduced candidate region for the sex-determining gene(s) and a set of tightly sex-linked SNP markers. Although we could not identify the causative gene(s), the fact that no female was mis-assigned using our sex-associated SNPs means that those SNPs could be also of high practical value towards the production of all male stocks for the *O. niloticus* aquaculture industry.

## Supporting Information

Table S1 **Sample origins and RAD barcodes.** Details each sample used: sample ID, family, gender, RAD barcode (index) used, number of raw reads (paired-ended or single-ended) and number of RAD-tags.
(CSV)

Table S2 **Genetic maps.** Ordered markers: marker ID, linkage group and position (cM).
(CSV)

Table S3 **KASP assay primer sequences.** List of the allele-specific primers and common primer designed for the allele-specific PCR genotyping assay of the five markers as well as their NCBI dbSNP accession numbers.
(CSV)

Table S4 **Details of the KASP assay results.** Genotypes of the 280 assays.
(CSV)

Data S1 **Marker sequences.** Details of SNP alleles and RAD-tag allele sequences of the five markers. (FASTA format)
(TXT)

Data S2 **Details of the physical location of the markers and the neighbouring annotated genes.** (GFF3 format)
(TXT)
