# Correction: Mapping and Validation of the Major Sex-Determining Region in Nile Tilapia (*Oreochromis niloticus* L.) Using RAD Sequencing

**DOI:** 10.1371/annotation/6320d31e-a818-445a-9c44-77a2ba5fe8e0

**Published:** 2013-09-09

**Authors:** Christos Palaiokostas, Michaël Bekaert, Mohd G. Q. Khan, John B. Taggart, Karim Gharbi, Brendan J. McAndrew, David J. Penman

In the author byline the first three authors, Christos Palaiokostas, Michaël Bekaert, and Mohd G. Q. Khan, should all be noted as equal contributors.

